# Supplementary material for: The Small RNA Universe of Capitella teleta
Source: Front Mol Biosci. 2022 Feb 25;9:802814. doi: 10.3389/fmolb.2022.802814 (PMC8915122; doi:10.3389/fmolb.2022.802814)
Supplement: Supplementary file 1 [file DataSheet1.ZIP › Supplement/known/CAPTEscaffold_434_21441.pdf]

Provisional ID	:	CAPTEscaffold_434_21441
Score total	:	47085
Score for star read(s)	:	3.9
Score for read counts	:	47078.9
Score for mfe	:	1.2
Score for randfold	:	1.6
Score for cons. seed	:	-0.6
Total read count	:	92355
Mature read count	:	92344
Loop read count	:	0
Star read count	:	11

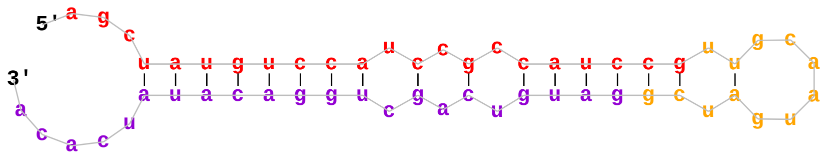

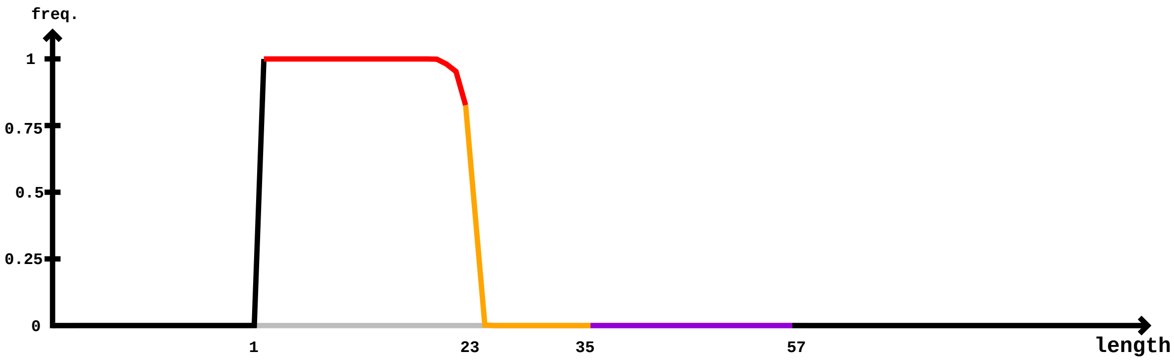

Mature **Star**

```
5'- uuauaggaaguagcuggcugagcuauguccauccgccauccguugcaaugaucggaugucagcuggacauaucacagaaagcucuucaagucgucuaacguaugugugcauc -3'  obs
    uuauaggaaguagcuggcugagcuauguccauccgccauccguugcaaugaucggaugucagcuggacauaucacagaaagcucuucaagucgucuaacguaugugugcauc      exp
    uuauaggaaguagcuggcugagcuauguccauccgccauccguugcaaugaucggaugucagcuggacauaucacagaaagcucuucaagucgucuaacguaugugugcauc      known
    ......((((.((((..(((.(.((((((((.(.(.((((((.(......).)))))).).).)))))))).).)))..))))))))......................... reads  mm  sample

    uuauaggaaguagcuggcug............................................................................................ 3     0   seq
    .uauaggaaguagcuggcug............................................................................................ 6     0   seq
    ..auaggaaguagcuggcug............................................................................................ 3     0   seq
    ...................Cagcuauguccauccgccaucc....................................................................... 1     1   seq
    ...................gagcuauguccauccgccauccgA..................................................................... 1     1   seq
    ....................agcuauguccauccgccU.......................................................................... 1     1   seq
    ....................agcuauguccauccgcca.......................................................................... 78    0   seq
    ....................Ngcuauguccauccgcca.......................................................................... 1     1   seq
    ....................agcuauguccauccgcAau......................................................................... 1     1   seq
    ....................agcuauUuccauccgccau......................................................................... 1     1   seq
    ....................agcuauguccauccgccaA......................................................................... 3     1   seq
    ....................agcuauguccaucUgccau......................................................................... 2     1   seq
    ....................Ggcuauguccauccgccau......................................................................... 11    1   seq
    ....................agcuaugAccauccgccau......................................................................... 1     1   seq
    ....................agcuGuguccauccgccau......................................................................... 1     1   seq
    ....................agcuaugGccauccgccau......................................................................... 3     1   seq
    ....................agcuauguUcauccgccau......................................................................... 2     1   seq
    ....................agcuauguccauccgccGu......................................................................... 3     1   seq
    ....................agcuaugucAauccgccau......................................................................... 2     1   seq
    ....................agcuaugucUauccgccau......................................................................... 2     1   seq
    ....................agcuauguGcauccgccau......................................................................... 1     1   seq
    ....................agcuauAuccauccgccau......................................................................... 1     1   seq
    ....................aCcuauguccauccgccau......................................................................... 1     1   seq
    ....................agcAauguccauccgccau......................................................................... 3     1   seq
    ....................agcuauguccauccgccau......................................................................... 1632  0   seq
    ....................agcuauguccauccgcUau......................................................................... 3     1   seq
    ....................agcuauguccaAccgccau......................................................................... 1     1   seq
    ....................agcuauguccauccgccaC......................................................................... 1     1   seq
    ....................Ngcuauguccauccgccau......................................................................... 3     1   seq
    ....................agcuauguccauccgccauU........................................................................ 229   1   seq
    ....................agcuauguccaucAgccauc........................................................................ 5     1   seq
```

Mature **Star**

uuauaggaaguagcuggcugagcuauguccauccgccauccguugcaaugaucggaugucagcuggacauaucacagaaagcucuucaagucgucuaacguaugugugcauc			
....................agcuauguccaAccgccauc..	1	1	seq
....................agcuauguUcauccgccauc..	1	1	seq
....................agcAauguccauccgccauc..	2	1	seq
....................agcuGuguccauccgccauc..	5	1	seq
....................agcuauguccauccgccauA..	6	1	seq
....................agcuaAguccauccgccauc..	2	1	seq
....................agcuaugAccauccgccauc..	2	1	seq
....................agcuaCguccauccgccauc..	1	1	seq
....................Ggcuauguccauccgccauc..	13	1	seq
....................agcuauguccGuccgccauc..	1	1	seq
....................agcuauguccauccgcUauc..	1	1	seq
....................agcuauguAcauccgccauc..	1	1	seq
....................agcuaugucUauccgccauc..	2	1	seq
....................agcuauguccauccgccauc..	2317	0	seq
....................agcuauguccaucUgccaucc...	43	1	seq
....................agcCauguccauccgccaucc...	2	1	seq
....................agcuauguccaCccgccaucc...	2	1	seq
....................agcuauguccauccgccauAc...	4	1	seq
....................aUcuauguccauccgccaucc...	1	1	seq
....................agcuauguccauccgccauUc...	3	1	seq
....................agcuauguccauccgAcaucc...	1	1	seq
....................agcuauguccauccgccauGc...	2	1	seq
....................agcuauguccauccgcAaucc...	2	1	seq
....................agcuauguccauccgccaucU...	49	1	seq
....................aNcuauguccauccgccaucc...	1	1	seq
....................agcuauguccauccgUcaucc...	2	1	seq
....................agcuauguccauccgcUaucc...	4	1	seq
....................agcuauguUcauccgccaucc...	2	1	seq
....................agcuaCguccauccgccaucc...	3	1	seq
....................agcuauguccauccgcGaucc...	2	1	seq
....................agcuauguAcauccgccaucc...	1	1	seq
....................agcuauguccaGccgccaucc...	1	1	seq
....................agcuauCuccauccgccaucc...	2	1	seq
....................agcuauguccauccUccaucc...	1	1	seq
....................agcuauguccauccgccaucG...	2	1	seq
....................aCcuauguccauccgccaucc...	2	1	seq
....................agcuauguccauccAccaucc...	5	1	seq
....................agcuGuguccauccgccaucc...	2	1	seq
....................agcuaugAccauccgccaucc...	5	1	seq
....................Ggcuauguccauccgccaucc...	53	1	seq
....................agcuaugucAauccgccaucc...	8	1	seq
....................agcuauAuccauccgccaucc...	3	1	seq
....................agcuaAguccauccgccaucc...	2	1	seq
....................agcuauguccauccCccaucc...	3	1	seq
....................agUuauguccauccgccaucc...	1	1	seq
....................agcuauguccaAccgccaucc...	9	1	seq
....................agcuauguccaucAgccaucc...	4	1	seq
....................agcuaugucUauccgccaucc...	18	1	seq
....................Ugcuauguccauccgccaucc...	2	1	seq
....................agcuauguccGuccgccaucc...	2	1	seq
....................agcuaugucGauccgccaucc...	1	1	seq
....................agcuauguccauccgccaucA...	9	1	seq
....................agcuauguccauGcgccaucc...	1	1	seq
....................agcuauguccauccgccaAcc...	2	1	seq
....................agcAauguccauccgccaucc...	10	1	seq
....................Ngcuauguccauccgccaucc...	4	1	seq
....................agcuauguccauccgccaucc...	11408	0	seq
....................aAcuauguccauccgccaucc...	2	1	seq
....................agAuauguccauccgccaucc...	3	1	seq
....................agcuauguccauccgccGucc...	5	1	seq
....................agcuauguccauAcgccaucc...	5	1	seq
....................agcuauUuccauccgccaucc...	1	1	seq
....................agcuauguccauccgccauAcg..	46	1	seq
....................agcuauguccauccgcUauccg..	42	1	seq
....................agcuaugucGauccgccauccg..	6	1	seq
....................aNcuauguccauccgccauccg..	2	1	seq
....................agcuauguccauccgccaucAg..	39	1	seq
....................agcuauguccauccgccaucGg..	2	1	seq
....................agcuauguccauccgccauccA..	119	1	seq
....................agcuauguccauccgccaCccg..	8	1	seq

Mature **Star**

uuauaggaaguagcuggcugagcuauguccauccgccauccguugcaaugaucggaugucagcuggacauaucacagaaagcucuucaagucgucuaacguaugugugcauc

....................agcuGuguccauccgccauccg..	7	1	seq
....................agcuauguccauccgccUuccg..	10	1	seq
....................agcuauguccauccgAcauccg..	8	1	seq
....................agcuauUuccauccgccauccg..	2	1	seq
....................agcuaGguccauccgccauccg..	3	1	seq
....................Ggcuauguccauccgccauccg..	626	1	seq
....................agcuauguccauccgccauGcg..	2	1	seq
....................agcuauguccaucUgccauccg..	87	1	seq
....................agcuauguUcauccgccauccg..	22	1	seq
....................agcuaugCccauccgccauccg..	14	1	seq
....................agcuauguccauccgccGuccg..	19	1	seq
....................agcuaugAccauccgccauccg..	41	1	seq
....................agcuauguccauccgccaucUg..	69	1	seq
....................agcuUuguccauccgccauccg..	2	1	seq
....................Ngcuauguccauccgccauccg..	16	1	seq
....................aAcuauguccauccgccauccg..	4	1	seq
....................agcuaAguccauccgccauccg..	37	1	seq
....................agcuaugucUauccgccauccg..	114	1	seq
....................agcuauguccauccgcGauccg..	10	1	seq
....................agcuauguccauGcgccauccg..	1	1	seq
....................agcuauguccauccgccauccg..	68585	0	seq
....................agcuaugucNauccgccauccg..	1	1	seq
....................agcuauguccaucAgccauccg..	43	1	seq
....................agcuauCuccauccgccauccg..	8	1	seq
....................agcuauguccauccCccauccg..	6	1	seq
....................agcuauguccauccgccauccC..	9	1	seq
....................agcuauguccauccgUcauccg..	6	1	seq
....................agcuauguccauccgccaGccg..	10	1	seq
....................agcuauguccauccgccauUcg..	6	1	seq
....................agcuaugGccauccgccauccg..	1	1	seq
....................agcuauguccauccUccauccg..	6	1	seq
....................agcuauguccaCccgccauccg..	14	1	seq
....................agcuauguccaAccgccauccg..	18	1	seq
....................agcuauguccauccgccauccU..	111	1	seq
....................agcuauguGcauccgccauccg..	8	1	seq
....................agcuauguccGuccgccauccg..	13	1	seq
....................agcuaCguccauccgccauccg..	9	1	seq
....................agcuauguccauccgcAauccg..	18	1	seq
....................agcuauguccaGccgccauccg..	1	1	seq
....................agcuauAuccauccgccauccg..	18	1	seq
....................agcuaugucAauccgccauccg..	63	1	seq
....................aUcuauguccauccgccauccg..	16	1	seq
....................Cgcuauguccauccgccauccg..	3	1	seq
....................agcuauguccauccAccauccg..	23	1	seq
....................aCcuauguccauccgccauccg..	5	1	seq
....................Ugcuauguccauccgccauccg..	5	1	seq
....................agcuauguccauccgccaAccg..	21	1	seq
....................agAuauguccauccgccauccg..	14	1	seq
....................agcAauguccauccgccauccg..	70	1	seq
....................agcuauguccauccgGcauccg..	6	1	seq
....................agUuauguccauccgccauccg..	2	1	seq
....................agcuauguccauAcgccauccg..	25	1	seq
....................agcCauguccauccgccauccg..	7	1	seq
....................agcuauguAcauccgccauccg..	20	1	seq
....................agcuauguccauUcgccauccg..	7	1	seq
....................agcuauguccUuccgccauccg..	7	1	seq
....................agcuauguccaucGgccauccg..	4	1	seq
....................Cgcuauguccauccgccauccgu...	1	1	seq
....................agcuauguAcauccgccauccgu...	1	1	seq
....................agcuauguccauccAccauccgu...	1	1	seq
....................Ggcuauguccauccgccauccgu...	45	1	seq
....................agcuauguccauccgccaucUgu...	4	1	seq
....................agcuauguccaucUgccauccgu...	3	1	seq
....................agcuaugucAauccgccauccgu...	2	1	seq
....................agcuauguccauccgccauccgN...	1	1	seq
....................agcuauguccaucAgccauccgu...	5	1	seq
....................agcuauguccauccgccauccAu...	9	1	seq
....................agcuauguccauccgccauccgC...	7	1	seq
....................agcuauguccauccgccauccgA...	1293	1	seq
....................agcuauguccauccgccGuccgu...	1	1	seq

```
                    Mature                            Star
uuauaggaaguagcuggcugagcuauguccauccgccauccguugcaaugaucggaugucagcuggacauaucacagaaagcucuucaagucgucuaacguaugugugcauc
....................agcuauguccauccgccauccgu.....................................................................    4113    0    seq
....................agcuauguccaCccgccauccgu.....................................................................    1       1    seq
....................agcuauguccauccgccaAccgu.....................................................................    2       1    seq
....................agcuaugucGuccgccauccgu.....................................................................    2       1    seq
....................agcuauguccauccgccauUcgu.....................................................................    1       1    seq
....................agcuauguccauccgAcauccgu.....................................................................    1       1    seq
....................agcuaugucUauccgccauccgu.....................................................................    5       1    seq
....................Ngcuauguccauccgccauccgu.....................................................................    1       1    seq
....................agcuauguUcauccgccauccgu.....................................................................    1       1    seq
....................agcuauguccauccgccauccgG.....................................................................    32      1    seq
....................agcAauguccauccgccauccgu.....................................................................    5       1    seq
....................agAuauguccauccgccauccgu.....................................................................    1       1    seq
....................agcGauguccauccgccauccgu.....................................................................    1       1    seq
....................Ugcuauguccauccgccauccgu.....................................................................    1       1    seq
....................agcuauguccauccgccauccUu.....................................................................    3       1    seq
....................agcuauguccauGcgccauccgu.....................................................................    1       1    seq
....................agcuauguccauccgccauAcgu.....................................................................    4       1    seq
....................agcuauguccauccgcUauccgu.....................................................................    2       1    seq
....................Ggcuauguccauccgccauccguu....................................................................    1       1    seq
....................agcuauguccauccgccauccUuu....................................................................    1       1    seq
....................agcuauguccauccgccauccCuu....................................................................    1       1    seq
....................agcuauguccauccgccauccguu....................................................................    176     0    seq
....................agcuauguccauccgccauccguA....................................................................    1       1    seq
....................agcuauguccauccgccauccgAu....................................................................    3       1    seq
....................agcuauguccauccgccauccguuU...................................................................    5       1    seq
.....................gcuauguccauccgccauccg......................................................................    1       0    seq
.....................gcuauguccauccgccauccgu.....................................................................    2       0    seq
......................cuauguccauccgccauccg......................................................................    2       0    seq
.......................uauguccauccgccauccg......................................................................    5       0    seq
.................................................gaucggaugucagcuggaca...........................................    1       0    seq
......................................................gaugucagcuggacauaucaca....................................    8       0    seq
......................................................gaugucGgcuggacauaucaca....................................    1       1    seq
.......................................................augucagcuggacauaucaca....................................    2       0    seq
```
